# Phagocytosis of the Lyme Disease Spirochete, *Borrelia burgdorferi*, by Cells from the Ticks, *Ixodes scapularis* and *Dermacentor andersoni*, Infected with An Endosymbiont, *Rickettsia peacockii*

**DOI:** 10.1673/031.007.5801

**Published:** 2007-11-20

**Authors:** Joshua T. Mattila, Ulrike G. Munderloh, Timothy J. Kurtti

**Affiliations:** Department of Entomology, University of Minnesota, St. Paul, MN 55108

**Keywords:** coiling phagocytosis, tick cell culture, fluorescence microscopy, live cell imaging, vector competency

## Abstract

Tick cell lines were used to model the effects of endosymbiont infection on phagocytic immune responses. The lines tested for their ability to phagocytose the Lyme disease spirochete, *Borrelia burgdorferi* (Spirochaetales: Spirochaetaceae), were ISE6 and IDE12 from the black-legged tick, *Ixodes scapularis* Say (Acari: Ixodidae) and DAE15 from the Rocky Mountain wood tick, *Dermacentor andersoni* Stiles. *Rickettsia peacockii* (Rickettsiales: Rickettsiaceae), an endosymbiont of *D. andersoni*, was used as a representative tick endosymbiont. 70–80% of uninfected or *R*. peacocciz-infected IDE12 and DAE15 cells phagocytosed heat-killed borreliae and 80–90% of IDE12 and DAE15 cells phagocytosed viable spirochetes. ISE6 cells were permissive of spirochetes; less than 1% of these cells phagocytosed borreliae, and spirochetes remained adherent to the cells seven days after inoculation. Cytochalasin B blocked phagocytosis of killed and viable borreliae by IDE12 cells, and prevented phagocytosis of killed spirochetes by DAE15 cells, whereas viable spirochetes successfully invaded cytochalasin-treated DAE15. IDE12 and DAE15 cells degraded borreliae within phagolysosome-like compartments. Time-lapse microscopy showed that DAE15 cells phagocytosed borreliae more rapidly than IDE12 cells. IDE12 and DAE15 cells eliminated most adherent spirochetes within 7 days of inoculation. Thus, endosymbiont infection does not significantly interfere with the phagocytic activity of immunocompetent tick cells.

## Introduction

Ticks protect themselves against bacterial pathogens with cellular and humoral immune responses that include phagocytosis and synthesis of antimicrobial peptides. Several tick tissues, including circulating blood cells called hemocytes, and the midgut, respond to bacteria with phagocytosis. Immunocompetent hemocytes have been reported to engulf *Borrelia burgdorferi* (Rittig et al. 1996; Coleman et al. 1997; Johns et al. 2000), *Micrococcus lysodeikticus* (Kuhn and Haug 1994), *Coxiella burnetii* (Bazlikova et al. 1984), and fungi including *Candida haemulonii* (Loosova et al. 2001). Moreover, the tick midgut uses endocytosis and phagocytosis to ingest components of the bloodmeal (Sonenshine 1991) and can phagocytose *Escherichia coli* (Matsuo et al. 2004), suggesting that the midgut is a functional component of the tick cellular immune system.

Ticks are infected with pathogenic and nonpathogenic rickettsia-like bacteria (Noda et al. 1997; Perlman et al. 2006) that may supply nutrients to the tick (Hotopp et al. 2006). Fortuitous intrahemocoelic bacterial contaminants are rare in ticks (Munderloh and Kurtti 1995), consequently, the bacteria present internally in ticks may have adapted to avoid the tick immune system and could potentially be infectious to vertebrate hosts. Given this, it has been suggested that the tick immune system has a role in determining the ability of ticks to transmit pathogenic bacteria to vertebrates (Johns et al. 2001). Unfortunately, little is known how endosymbionts impact tick physiology and interactions between the tick immune system and endosymbiotic bacteria remain to be characterized.

We used tick cell culture to model the effect of endosymbionts on the phagocytic response of tick cells using the Lyme disease spirochete, *Borrelia burgdorferi* (Spirochaetales: Spirochaetaceae), a tick-transmitted bacterium, as the target of phagocytosis. Endosymbionts of *Ixodes scapularis* were not available and so *Rickettsia peacockii* (Rickettsiales: Rickettsiaceae), a nonpathogenic bacterium from the Rocky Mountain wood tick, *Dermacentor andersoni* Stiles (Acari: Ixodidae) (Niebylski et al. 1997), was used as a representative tick endosymbiont. Additionally, cellular factors influencing vector competency were examined by comparing the responses of two cell lines from the black-legged tick, *Ixodes scapularis* Say (Acari: Ixodidae), the normal vector of *B. burgdorferi*, with a line from *D. andersoni*, a tick not known to vector *B. burgdorferi* (Dolan et al. 1997). The *I. scapularis* ISE6 line did not phagocytose borreliae and the spirochetes survived and were able to multiply. In contrast, the *I. scapularis* line IDE12 and *D. andersoni* line DAE15 were phagocytic, and killed borreliae in phagolysosome-like compartments. Cellular motility, which was greatest in the DAE15 line, was an important factor determining the rate at which these cell lines eliminated spirochetes. We interpret these results to indicate that differences in the responsiveness of tissues involved in cellular immune responses may be a determinant of vector competency in ticks. *R. peacockii* infection minimally inhibited phagocytosis of viable spirochetes by DAE15 cells, but not by IDE12 cells. Moreover, infection did not modify elimination of adherent borreliae, or degradation of phagocytosed borreliae, by DAE15 or IDE12 cells. These results suggest that infection with rickettsia-like endosymbionts is unlikely to interfere with the ability of tick cells to phagocytose and kill bacteria.

## Materials and Methods

### Spirochete culture and labeling

Low passage *B. burgdorferi* isolate JMNT (Kurtti et al. 1988) and KS20 (Babb et al. 2004), a GFP-expressing strain of the *B. burgdorferi* isolate B31, were grown in Barbour-Stoenner-Kelly medium (Barbour 1984) supplemented with 6% heat-inactivated rabbit serum (Gibco, www.lifetech.com) and 10% gelatin (Difco, www.voigtglobal.com/DIFCO.htm). Cultures were maintained at 34°C and passaged during mid-log growth phase by diluting 1 ml of spirochete-containing medium into 5 ml of fresh medium. JMNT borreliae were centrifuged at 3,000 × g for 15 minutes and the pellet washed once with Hank's balanced saline solution (Invitrogen, www.invitrogen.com). Spirochetes were killed by resuspending the pellet in 5 ml of Hanks' solution pre-heated to 55°C, and incubated at 55°C for 20 minutes. Loss of viability was confirmed with the BacLight Bacterial Live/Dead kit (Molecular Probes, www.probes.com). Heat-killed bacteria were labeled with a 1 mg/ml solution of fluorescein isothiocyanate isomer I (FITC) (Sigma, www.sigmaaldrich.com) in Hanks' saline for 30 minutes on a rocking plate at room temperature. Bacteria were centrifuged at 21,000 × g for 5 minutes and the pellet washed several times in Hanks' saline to remove unbound FITC. Heat killed FITC-labeled *B. burgdorferi* (hkFITC-Bb) were examined by fluorescence microscopy to ensure adequate labeling and maintenance of normal spirochetal morphology. HkFITC-Bb were counted by diluting 1 µl bacterial stock into 4 µl VectaShield mounting medium containing 4′-6-Diamidino-2-phenylindole (DAPI-VectaShield; Vector Laboratories, www.vectorlabs.com), and counted with a Petroff-Hauser hemocytometer under epifluorescence. Bacterial stocks were kept in the dark at 4°C until used.

### Tick cell culture

The *I. scapularis* lines IDE12 (Munderloh et al. 1994), ISE6 (Obonyo et al. 1999) and the *D. andersoni* line DAE15 (Kurtti et al. 2005), were grown in 25 cm^2^ CellStar tissue culture flasks (Greiner Bio-One Inc, www.gbo.com) containing 5 ml L15B (Munderloh and Kurtti 1989) supplemented with 5% fetal bovine serum, 5% tryptose phosphate broth (Becton Dickinson, www.bd.com), and 0.1% cholesterol concentrate (MP Biomedicals, www.mpbio.com). Cultures were maintained at 34°C and fed fresh medium weekly. Cultures challenged with borreliae were fed medium supplemented with 10% Barbour-Stoenner-Kelly medium to accommodate spirochete nutritive requirements. Negative effects on tick cells were not observed in cultures maintained on this medium. Cultures were infected with host-cell free preparations of *R. peacockii* made by filtering medium from heavily infected ISE6 cultures containing cell-free rickettsiae through a 5.0 µm syringe filter (Millex-SV; Millipore, www.waters.com) and adding the rickettsiae to established cultures. The infection status of cultures was monitored by comparing the proportion of infected to uninfected cells in Giemsa-stained Cytospin preparations (Shandon, www.thermo.com).

For plate-based assays, tick cells were resuspended in fresh culture medium, counted with a hemocytometer, and adjusted to a density of 1×10^5^ cells/ml, 1 ml of cell suspension (1×10^5^ cells) was seeded into a sterile 24 well tissue culture plate (Corning, www.corning.com/lifesciences) containing sterile 15 mm diameter circular glass coverslips (Electron Microscopy Services, www.2spi.com) and given 5 days to adhere and recover from subculturing at 34°C before bacterial challenge.

### Phagocytosis assay with hkFITC-Bb

Cell layers in 24 well tissue culture plates were examined by phase contrast microscopy before assay to ensure the cells were adherent and had normal morphology. Old medium was removed and half the wells were filled with 0.5 ml of fresh medium. The other half were filled with 0.5 ml medium containing 5 µg/ml cytochalasin B (CB) (Sigma) and incubated for 30 minutes at 34°C. CB depolymerizes actin causing distinct morphological changes including loss of pseudopodia. Cultures were examined by phase contrast microscopy prior to challenge to ensure cell morphology was altered and the cells had the phenotype indicative of actin depolymerization. Live JMNT borreliae and hkFITC-Bb were counted, centrifuged at 3,000 × g for 15 minutes, and resuspended in fresh L15B at 4×10^6^ spirochetes/ml and each well given 0.5 ml for a multiplicity of infection of 20 bacteria/cell. Plates were incubated overnight at 34°C. Culture medium was removed and coverslips were washed with PBS to remove unbound spirochetes. Cell layers were fixed with a 20 minute incubation in 2% paraformaldehyde (EMD Chemicals, Inc., www.emdchemicals.com) in PBS, washed once with PBS, and counterstained with 5.2 µM Evan's blue (EB) (Sigma) in PBS for 5 minutes. Excess stain was removed with two washes of PBS and the coverslips were removed and briefly air dried. Coverslips were mounted on slides with 4.2 µl DAPI-VectaShield. Slides were viewed with a Nikon Eclipse E400 fluorescence microscope (Nikon, www.nikon.com) using filter sets for FITC, DAPI, and Texas Red/FITC. Digital images were acquired with a DMX-1200 digital camera (Nikon), and one image acquired for each filter set. Data represent the mean ± standard deviation of three independent experiments.

### Phagocytosis assay for viable JMNT borreliae

Cell layers to be challenged with live borreliae were established on the same plate as cells to be challenged with hkFITC-Bb. Cells were treated and washed as previously described. Coverslips were left in the wells and fixed, then incubated for 1 hour in 3% bovine serum albumin in PBS (BSA-PBS) at room temperature. BSA-PBS was removed and replaced with a 1:50 dilution of BacTrace® anti-*B. burgdorferi* FITC-conjugated polyclonal antibody (KPL, www.kpl.com) and incubated for 30 minutes at 37°C. Unbound antibody was removed by two washes with PBS. Coverslips were fixed for 5 min in absolute methanol, removed and briefly air dried before being mounted on slides with DAPI-VectaShield. Cells were examined by fluorescence microscopy and determined to be phagocytic when they contained uniformly coiled, DAPI-stained borreliae within the borders of the cell. Rickettsial infection was confirmed by viewing the DAPI-stained stained cells. At least 300 cells per coverslip were counted, and three replicates per treatment were evaluated for at least 1000 cells/experiment. Data are expressed as the mean of three experiments. Statistical analysis was done using SigmaStat software (Systat Software, Inc., www.systat.com).

### Time lapse fluorescence microscopy and localization of ingested borrelia to lysosomes

Cells were seeded into Glass Bottom Microwell Dishes (MatTek Corporation, www.glass-bottom-dishes.com) and allowed to grow 5 days before being challenged with KS20 borrelia. Cultures were stained with 60 nM LysoTracker Red DND-99 (LTR) (Molecular Probes, www.probes.com), a cell permeant stain that is protonated in an acidic compartment, becoming fluorescent and membrane impermeant (Yeung et al. 2005), to further characterize the conditions that spirochetes were exposed to following phagocytosis. Cell layers were washed with PBS, and incubated with 60 nM LTR for 30 minutes at 32°C to visualize lysosomes. Cell layers were washed twice with PBS to remove extra stain, borreliae were added (multiplicity of infection = 20) and incubated 30 minutes at 32°C to allow borreliae to settle onto the cells. Time lapse fluorescence microscopy was done on a Nikon TE2000-U microscope using an oil immersion 40× objective. Z-series of green and red fluorescent images was taken every 30s for 90 minutes for DAE15 cells or 105 minutes for IDE12 cells. Images were acquired and assembled using MetaMorph software (Molecular Devices, www.moleculardevices.com). Overnight incubation was performed to examine the fate of phagocytosed spirochetes and acidified organelles were stained with LTR and visualized by fluorescence microscopy with filters for Texas Red/FITC and DAPI. Tick cells were incubated overnight with KS20 borreliae as previously described for the live borreliae phagocytosis assays. Cells were washed with PBS, and incubated with 60 nM LTR in PBS at 34°C for 30 minutes. Cells were washed 2 times with PBS and fixed for 20 minutes in 2% paraformaldehyde (EMS). Coverslips were washed with PBS and mounted on slides as previously described. LTR staining was confirmed with LysoTracker Green (Molecular Probes) using the same procedure.

### Measuring loss of viability with GFP-expressing borreliae

Cells were grown as previously described for live JMNT borreliae phagocytosis assays. Prolonged incubation with CB caused cell layers lift off the substrate, and was not used in these experiments. Viable KS20 borreliae were added to the cells and incubated for 1, 2, 5, and 7 days. Cells and borreliae were fixed and counterstained as described for the hkFITC-Bb phagocytosis assay. Cell layers were examined at 100 × magnification, and four factors were assessed per field: total number of cells, cells containing coiled spirochetes, cells containing coiled spirochetes with residual GFP expression, and cells having morphologically normal adherent GFP-positive KS20 borreliae. At least 100 cells were evaluated per coverslip, with coverslips examined from three replicate wells per time point. Data are expressed as the mean percent cells ± standard deviation of three replicate experiments.

## Results

### Infection of *I. scapularis* and *D. andersoni* cell lines with *R. peacockii*

The cell lines were used to model tick tissues, and were morphologically distinct from each other. The DAE15 line was composed mostly of rounded cells, but also contained flattened, macrophage-like cells. IDE12 cultures were heterogeneous populations of rounded cells, cells having numerous filipodia, and flattened macrophage-like cells. The morphologies of cells in the ISE6 line were more uniform, with the majority of cells having neuron-like appearances with finely branching pseudopodia.

Different responses to infection by *R. peacockii* were observed. The ISE6 line rapidly attained a high proportion of infected cells, and heavily infected cell layers would lift off the flask or coverslip. Consequently, infection of ISE6 cells was maintained by transferring infected cells to uninfected cell layers. In contrast, the *R. peacockii*-infected IDE12 and DAE15 cell lines could be passaged in a persistently infected state. It was not possible to uniformly infect the cell layers and cultures contained infected cells with estimated numbers of *R. peacockii* ranging from two to three bacteria per cell to several hundred per cell. The mean proportion of infected cells in DAE15 cultures was 83.0% (range 72.0–93,0%) and for IDE12 cells was 61.3% (range 45.0–83.1%).

**Figure 1.**
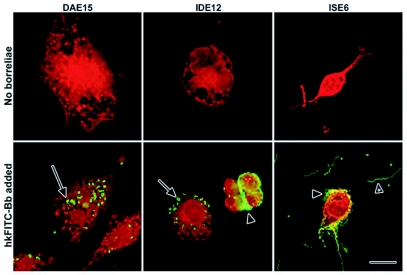
Response of tick cells to challenge with heat-killed FITC-labeled JMNT *Borrelia burgdorferi*. Cells were fixed and stained with Evan's blue and photographed at 50× magnification. Clusters of extracellular adherent borreliae are indicated by arrowheads. Note the withdrawn pseudopodia and borreliae coating the ISE6 cell. Unbound spirochetes can be seen in the area surrounding the ISE6 cell (arrowhead with asterisk). Scale bar 20 µm.

### Phagocytosis of *B. burgdorferi*

ISE6 cell layers incubated with viable JMNT *B. burgdorferi*, resuspended and stained with Giemsa, had numerous bacteria adhering to the cell body and pseudopodia, while IDE12 and DAE15 cells had dramatically fewer adherent spirochetes, but contained numerous spirochetes that were coiled inside vacuoles (not shown).

**Figure 2.**
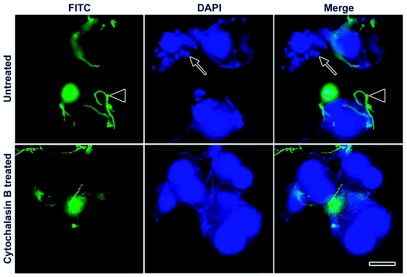
Phagocytosis of live *Borrelia burgdorferi* by IDE12. IDE12 cells were incubated with viable borreliae for 24 hours, fixed, and the external borreliae stained with a FITC-conjugated anti-borrelia antibody. Phagocytosis by untreated cells was compared with cells treated with cytochalasin B, an inhibitor of phagocytosis. External, morphologically normal borreliae are adherent to cells in the FITC panel (arrowheads), while external and internal spirochetes and cell nuclei can be seen in the DAPI panel. Coiled spirochetes are seen inside the cells (arrows). Scale bar, 10 µm.

IDE12 and DAE15 cells incubated with heat-killed fluorescein (FITC)-labeled JMNT borreliae (hkFITC-Bb) contained numerous intracellular coiled borreliae while ISE6 cells had large numbers of adherent borreliae (Figure 1), Confocal microscopy was used to demonstrate the intracellular localization of spirochetes within vacuoles (not shown) and intracellular localization was confirmed with double labeling experiments using FITC-conjugated anti-borrelia polyclonal antibodies and 4′-6-Diamidino-2-phenylindole (DAPI) (Figure 2), In these experiments, extracellular spirochetes stained with the FITC-conjugated antibodies, whereas the intracellular and extracellular spirochetes stained with DAPI. Cell nuclei also stained with DAPI. The coiled conformation of intracellular spirochetes (Figure 2, arrow) was used as an indicator of phagocytosis.

**Figure 3.**
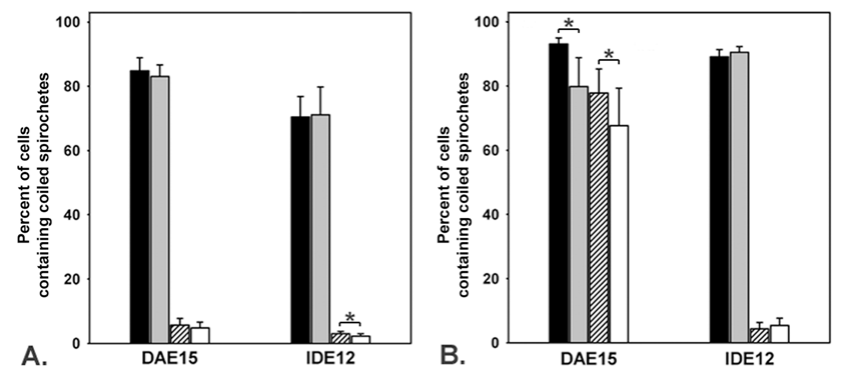
Phagocytosis of borrelia by DAE15 and IDE12 cells. Cell layers were incubated with hkFITC or viable JMNT borreliae for 24 hours prior to fixation and examination. Cells containing internalized spirochetes with a coiled appearance were considered phagocytic. Phagocytosis of borrelia by uninfected cells (black bars) and *Rickettsia peacockii*-infected cells (grey) were compared uninfected cells treated with cytochalasin B (crosshatched) and *R. peacockii*-infected cells treated with cytochalasin B (white) cultures. Asterisks indicated statistically significant differences between samples. A. Phagocytosis of dead *Borrelia burgdorferi* by DAE15 and IDE12 cells. B. Phagocytosis of live borreliae by DAE15 and IDE12 cells.

**Figure 4.**
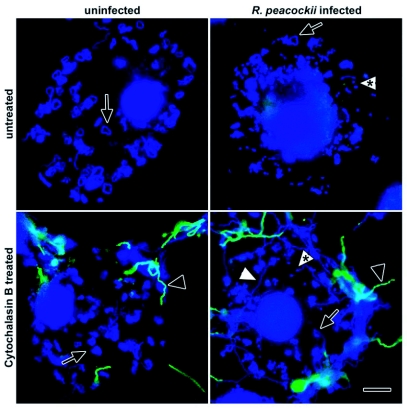
Phagocytosis of live *Borrelia burgdorferi* by uninfected and *Rickettsia peacockii*-infected DAE15 cells. Coiled spirochetes are seen inside the cells (arrows) and morphologically-normal spirochetes stained with FITC-conjugated polyclonal antiborrelia antibodies can be seen adherent to cytochalasin B-treated cells (black arrowheads). Notice that morphologically normal spirochetes (white arrowheads) and coiled spirochetes are also present in the cytochalasin B treated cells. *R. peacockii* are indicated by the arrowheads with asterisks. Scale bar, 10 µm.

Cultures were challenged with hkFITC-Bb or living borreliae as described in the experimental procedures. Replicate cultures were incubated with cytochalasin B (CB) to inhibit phagocytosis. CB treatment did not visibly affect spirochete motility or morphology. Phagocytic capacity was determined by comparing the number of cells containing coiled borreliae with the number of cells without coiled borreliae. Infection by *R. peacockii* did not influence the ability of DAE15 or IDE12 cells to phagocytose heat killed spirochetes (Figure 3A). CB effectively inhibited phagocytosis of hkFITC-Bb by DAE15 and IDE12 cells. Differences in the numbers of phagocytic cells between CB-treated uninfected and infected IDE12 cells were statistically significant (t = 2,248, df = 16, p < 0.05). A greater proportion of uninfected and *R*. peacocfcii-infected IDE12 cells phagocytosed living borreliae, and similar to what was observed for IDE12 cells incubated with hkFITC-Bb, CB almost completely inhibited phagocytosis of living borreliae. A greater proportion of DAE15 cells were phagocytic when challenged with viable borreliae (Figure 3B). Rickettsial infection did, however, diminish the number of DAE15 cells containing coiled spirochetes relative to the number of uninfected cells containing coiled spirochetes (t = 2.191, df = 16, p < 0.05). Unexpectedly, uninfected and *R. peacockii*-infected DAE15 cells treated with CB contained coiled spirochetes (Figure 4).

### Phagocytosis of live *B. burgdorferi*; elimination of adherent borreliae and intracellular killing

Preliminary experiments showed KS20 spirochetes retained GFP fluorescence after fixation with 2% paraformaldehyde but not 70% ethanol, indicating GFP fluorescence was lost by membrane permeabilization or degradation of the GFP fluorophore. Consequently, we used fluorescence of KS20 spirochetes as an indicator of membrane integrity and bacterial viability. Declining numbers of cells with morphologically-normal spirochetes (elimination of adherent borreliae) was considered an indicator of phagocytosis. Phagocytosed spirochetes were seen coiled in the cytoplasm and those retaining GFP fluorescence were considered intact. Those that did not retain GFP fluorescence, and only stained with DAPI, were considered degraded.

**Figure 5.**
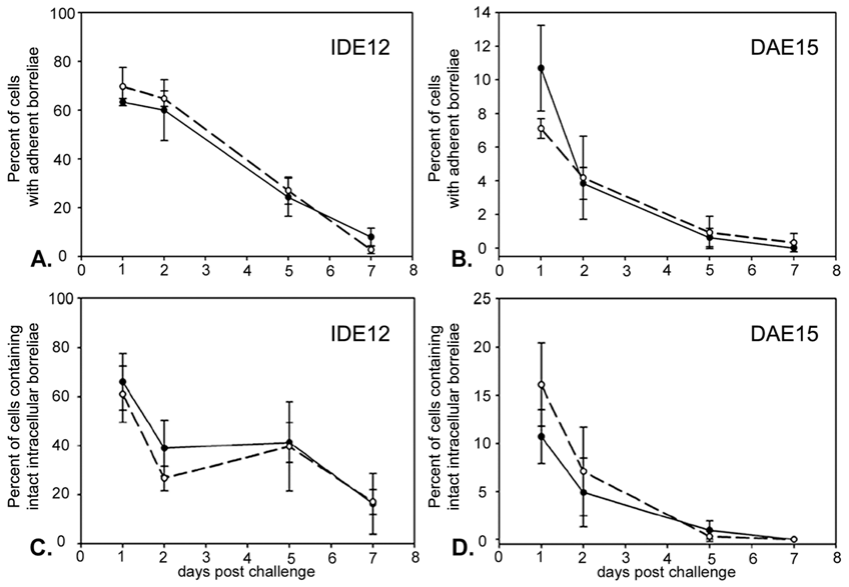
Adherent GFP-expressing borreliae are ingested and degraded following cocultivation with IDE12 and DAE15 cells. Uninfected cells are indicated by solid line and closed circles, *Rickettsia peacockii*-infected cells by dashed line and open circles. Differences in the number of uninfected or *R. peacockii*-infected cells with adherent or phagocytosed GFP-retentive borreliae are not statistically significant at any time point. A. Percent of uninfected and *R. peczcockii*-infected IDE12 cells with adherent borreliae. B. Percent of uninfected and *R. peacockii*-infected DAE15 cells with adherent borreliae. C. Percent of uninfected and *R. peacockii*-infected IDE12 containing phagocytosed but intact borreliae. D. Percent of uninfected and *R. peacockii*-infected DAE15 containing phagocytosed but intact borreliae.

**Figure 6.**
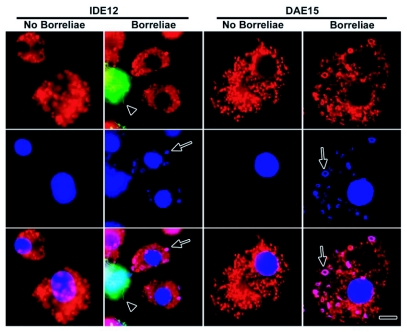
Colocalization of phagocytosed GFP-expressing KS20 borrelia with LysoTracker Red-stained phagolysosomes in IDE12 and DAE15 cells. IDE12 and DAE15 cells were incubated overnight with GFP-expressing KS20 borreliae, stained with LysoTracker Red, fixed with paraformaldehyde, and counterstained with DAPI to stain the DNA of spirochetes and cell nuclei. Phagocytosed borreliae are indicated by arrows. Note the loss of GFP fluorescence by phagocytosed the spirochetes (arrows) whereas GFP fluorescence is retained by extracellular spirochetes (arrowhead). Scale bar, 10 µm.

Spirochetes adhered to ISE6 cells throughout the 7-day study period, but less than 1% of the cells contained coiled GFP-expressing KS20 borreliae at 7 days post challenge (not shown). In contrast, the number of IDE12 and DAE15 cells with adherent borreliae declined over the same time period (Figures 5A and 5B). Infection with *R. peacockii* did not appreciably change the proportion of cells with adherent spirochetes. IDE12 cells did not completely eliminate adherent borreliae and 16.3 ± 12.3% of uninfected, and 17.1 ± 5.1% of *R. peacockii-fectea* cells had adherent spirochetes seven days post challenge. DAE15 cells eliminated spirochetes more rapidly, and less than one percent of uninfected or *R. peacockii-infectea* DAE15 cells had adherent spirochetes five days post challenge. The number of cells containing phagocytosed, but intact, borreliae decreased over the same time period (Figure 5C, 5D). IDE12 cells did not degrade all the phagocytosed spirochetes over seven days, and 8.0 ± 3.6% of uninfected cells and 2.7 ± 1.5% of *R. peacockii-infectea* IDE12 cells contained intact spirochetes. DAE15 cells degraded borreliae more effectively than IDE15 cells and less than 1% of uninfected and *R. peacockii*-infected cells contained phagocytosed, but intact, spirochetes after seven days. LysoTracker Red (LTR) staining showed phagocytosed spirochetes were digested in acidified compartments (Figure 6).

### Killing and intracellular processing of phagocytosed GFP-expressing KS20 spirochetes

Interactions between tick cells and KS20 borreliae were visualized by time lapse fluorescence microscopy. Motile spirochetes bound to IDE12 and DAE15 cells, lost normal spirochetal mobility and appeared to be coiled up by the cells. Soon after losing mobility, coiled spirochetes were transported towards the LTR-stained region of the cell and GFP fluorescence was lost within two minutes. DAE15 cells were motile and rapidly bound and phagocytosed spirochetes (Video clip 1). IDE12 cells were less mobile and required significantly more time to internalize and kill spirochetes relative to the DAE15 cells (Video clip 2). Additionally, fewer IDE12 cells were phagocytic when compared to the proportion of phagocytic DAE15 cells. Within 105 minutes following challenge, 5.6% (2 of 36) of IDE12 cells were observed to phagocytose spirochetes, whereas 47% (17 of 36) of DAE15 cells were phagocytically active over the same challenge period.

## Discussion

Interactions between arthropod vectors, endosymbiotic bacteria, and bacterial pathogens, may have important consequences on pathogen transmission as bacteria that can avoid the arthropod immune system may be able to overcome the vertebrate immune system (Waterfield et al. 2004). Many factors underlying vector capacity in ticks are not known, but the tick immune system may be an important determinant of which bacteria a tick may transmit (Johns et al. 2001). This hypothesis underscores the importance of clarifying how tick endosymbionts interact with the tick immune system, and understanding how immune responses to bacterial pathogens differ between vector and nonvector ticks.

Differences in the response of ticks to borreliae have been examined in *D. vanablis*, a tick that does not vector *B. burgdorferi*, and *I. scapularis*, a normal Lyme disease vector. Johns et al (2001; 2000) found *D. variablis* rapidly eliminated and killed *B. burgdorferi* that had been inoculated into the hemocoel. In contrast, borreliae inoculated into *I. scapularis* were eliminated much more slowly and morphologically normal spirochetes could be seen adhering to salivary glands and ovaries 24 hours post challenge (Johns et al. 2001). We observed a similar range of events in *vitro* when *I. scapularis* and *D. andersoni* cell lines were challenged with borreliae.

The ISE6 line from *I. scapularis* showed a very limited cellular response to spirochetal challenge. Heat inactivated and viable borreliae adhered tightly to surfaces of ISE6 cells, leading the cells to shorten and withdraw pseudopodia, but this was the extent of the ISE6 cellular response to borreliae. Very little phagocytosis was observed despite the close cell-borreliae association and the proportion of ISE6 cells associated with morphologically normal borreliae did not change over seven days. The tick cell lines described in this report are derived from embryonic tissues and the tissue types represented in culture are not known. Coiling phagocytosis is performed by cells dedicated to phagocytes (Rittig et al. 1996), suggesting either that the embryonic tissue this line is derived from was not phagocytically competent, or this ability has been lost during culture. ISE6 cultures have been used to isolate and cultivate borreliae (Obonyo et al. 1999), and these data reinforce studies indicating that ISE6 cells do not mount immune responses against borreliae.

In contrast to the permissiveness exhibited by ISE6 line, the IDE12 line from *I. scapularis*, and DAE15 line from *D. andersoni*, responded aggressively to challenge. IDE12 and DAE15 cells used coiling phagocytosis to ingest heat inactivated and viable borreliae. Fluorescence was lost by phagocytosed GFP-expressing spirochetes once they colocalized with the lysosome-rich region in IDE12 and DAE15 cells, suggesting that the bacterial membrane had been punctured and lysosomal factors degraded or denatured the GFP molecule. Loss of fluorescence occurred at the same rate in both lines, indicating the phagolysosomes of IDE12 and DAE15 cells contained similar effector molecules. IDE12 cells eliminated adherent borreliae more slowly than DAE15 cells, however, and required more time to ingest adherent spirochetes. Time lapse microscopy showed that DAE15 cells rapidly responded to bacterial challenge with increased motility and large extended pseudopodia, whereas IDE12 cells produced smaller pseudopodia and were more sedentary (not shown).

A greater proportion of cells challenged with viable borreliae contained coiled spirochetes relative to the proportion of cells incubated with heat-killed spirochetes. Additionally, DAPI-stained cells had morphologically-normal spirochetes that appeared to be inside or underneath the cells. Viable spirochetes may be more attractive targets for phagocytosis than heat-killed spirochetes and a proportion of cells may have been invaded by viable spirochetes. Spirochetes can seek intracellular environments to avoid antibiotics or antimicrobial peptides (Ma et al. 1991; Singh and Girschick 2004). The mechanisms by which borreliae penetrate cells are unclear, but interaction with the actin cytoskeleton appears to be required for invasion of mammalian cell lines (Ma et al. 1991). Kurtti et al (1994) observed ends of borreliae associated with clathrin-coated pits on the surface of cultured *Rhipicephalus appendiculatus* cells, suggesting that borreliae use actin dependent processes such as receptor-mediated endocytosis to invade tick cells. Interestingly, actin depolymerization did little to inhibit the proportion of DAE15 cells challenged with viable spirochetes that contained coiled borreliae even though cytochalasin B treatment almost completely inhibited phagocytosis of dead borreliae. Several bacteria, including *Actinobacillus actinomycetemcomitans*, an agent of periodontal disease, and the spirochete *Leptospira interrogeans*, use actin dependent and actin independent, microtubule-based mechanisms to invade mammalian cells (Merien et al. 1997; Brissette and Fives-Taylor 1999; Meyer et al. 1999). *B. burgdorferi* may be able to exploit similar mechanisms to invade tick cells. Invasion may not require a specific receptor-based mechanism because depolymerization of the cortical actin cytoskeleton could enable mechanical penetration of the plasma membrane.

There is little information on how endosymbionts affect tick immune responses. Tick cell culture can be used to model outcomes of tick-endosymbiont-microbe immune interactions that are difficult to examine in persistently infected ticks. Significant differences were not observed in the responses of uninfected or infected DAE15 cells to heat inactivated borreliae. Fewer *R. peacockii*-infected DAE15 cells phagocytosed viable borreliae than did uninfected DAE15 cells, DAE15 cells support heavy loads of rickettsiae and diminished phagocytosis in infected cells suggests that large pockets of rickettsiae physically limit normal motility or pseudopod formation. Rickettsial infection did not, however, significantly inhibit the rate at which cells cleared adherent borreliae or degraded borreliae. DAE15 cells were highly motile, and the diminished phagocytic capacity of highly infected cells may be compensated by lightly infected cells with greater motility ingesting more borreliae. IDE12 cells supported fewer rickettsiae per cell than DAE15 cells and may be a better model for the response of lightly infected tissues to bacterial challenge. *R. peacockii* infected IDE12 cells were not affected by rickettsial infection and had phagocytic capacities that were comparable to uninfected cells. Similar capacities to eliminate adherent borreliae and degrade phagocytosed borreliae were seen in uninfected and infected cells. Rickettsial peptidoglycan, which is similar to peptidoglycan from other gram-negative bacteria (Pang and Winkler 1994), did not activate immune responses in IDE12 cells. Moreover, rickettsial infection did not impact the ability of these cells to kill or degrade phagocytosed borreliae, suggesting the intracellular resources used to manage rickettsial populations do not interfere with degradation of phagocytosed microbes.

In conclusion, different responses to challenge by *B. burgdorferi* were noted between the tick cell lines, and whether the cells were infected with *R. peacockii*. ISE6 cells from *I. scapularis* were not phagocytic for borreliae. The cell line IDE12 from *I. scapularis*, and DAE15 from *D. andersoni*, phagocytosed spirochetes, and degraded them in phagolysosome-like vacuoles. A large proportion of DAE15 cells were invaded by viable *B. burgdorferi*. Infection by *R. peacockii* slightly inhibited phagocytosis of viable borreliae by DAE15 cells, but did not appear to affect phagocytosis by IDE12 cells. These data suggest differences in vector competency in ticks may be tied to cellular responses following bacterial challenge. Furthermore, the rickettsia-like endosymbionts that infect ticks are unlikely to significantly modify cellular immune responses, such as phagocytosis, to bacterial challenge.

## Footnotes

**Video Clip 1. :** This video can be accessed at the following URI: http://digital.library.wisc.edu/1793/22053

**Video Clip 2. :** This video can be accessed at the following URI: http://digital.library.wisc.edu/1793/22053

## **Abbreviations**

JMNT and KS20 -: *Borrelia burgdorferi* isolates
KS20 - a: *Borrelia burgdorferi* isolate
FITC -: fluorescein isothiocyanate isomer I
hkFITC-Bb -: heat killed FITC-labeled *Borrelia burgdorferi*
IDE12 and ISE6 -: *Ixodes scapularis* lines, DAE15 - *Dermacentor andersoni* line
